# Heme Oxygenase-1 and 2 Common Genetic Variants and Risk for Restless Legs Syndrome

**DOI:** 10.1097/MD.0000000000001448

**Published:** 2015-08-28

**Authors:** Elena García-Martín, Félix Javier Jiménez-Jiménez, Hortensia Alonso-Navarro, Carmen Martínez, Martín Zurdo, Laura Turpín-Fenoll, Jorge Millán-Pascual, Teresa Adeva-Bartolomé, Esther Cubo, Francisco Navacerrada, Ana Rojo-Sebastián, Lluisa Rubio, Sara Ortega-Cubero, Pau Pastor, Marisol Calleja, José Francisco Plaza-Nieto, Belén Pilo-de-la-Fuente, Margarita Arroyo-Solera, Esteban García-Albea, José A.G. Agúndez

**Affiliations:** From the Department of Pharmacology, Universidad de Extremadura, Cáceres, Spain (EG-M, JAGA); Section of Neurology, Hospital Universitario del Sureste, Arganda del Rey (Madrid), Spain (FJJ-J, HA-N, FN, MC, JFPN, BP-D-LF, MA-S); Department of Medicine-Neurology, Hospital “Príncipe de Asturias”. Universidad de Alcalá, Alcalá de Henares (Madrid), Spain (FJJ-J, HA-N, AR-S, LR, EG-A); Department of Pharmacology, University of Extremadura, Badajoz, Spain (CM); Section of Neurology, Hospital Virgen del Puerto, Plasencia (Cáceres), Spain (MZ); Section of Neurology, Hospital La Mancha-Centro, Alcázar de San Juan (Ciudad Real), Spain (LT-F, JM-P); Unit of Neurology, Clínica Recoletas, Zamora, Spain (TA-B); Section of Neurology, Hospital Universitario de Burgos, Burgos, Spain (EC); CIBERNED, Centro de Investigación Biomédica en Red de Enfermedades Neurodegenerativas, Instituto de Salud Carlos III, Spain (SO-C, PP); Neurogenetics Laboratory, Division of Neurosciences, Center for Applied Medical Research, Universidad de Navarra, Pamplona, Spain (SO-C, PP); Department of Neurology, Clínica Universidad de Navarra, University of Navarra School of Medicine, Pamplona, Spain (PP); and Department of Neurology, Hospital Universitari Mutua de Terrassa, Terrassa, Barcelona, Spain (PP).

## Abstract

Several neurochemical, neuropathological, neuroimaging, and experimental data, suggest that iron deficiency plays an important role in the pathophysiology of restless legs syndrome (RLS). Heme-oxygenases (HMOX) are an important defensive mechanism against oxidative stress, mainly through the degradation of heme to biliverdin, free iron, and carbon monoxide. We analyzed whether *HMOX1* and *HMOX2* genes are related with the risk to develop RLS.

We analyzed the distribution of genotypes and allelic frequencies of the *HMOX1* rs2071746, *HMOX1* rs2071747, *HMOX2* rs2270363, and *HMOX2* rs1051308 SNPs, as well as the presence of Copy number variations (CNVs) of these genes in 205 subjects RLS and 445 healthy controls.

The frequencies of *rs2071746TT* genotype and *rs2071746T* allelic variant were significantly lower in RLS patients than that in controls, although the other 3 studied SNPs did not differ between RLS patients and controls. None of the studied polymorphisms influenced the disease onset, severity of RLS, family history of RLS, serum ferritin levels, or response to dopaminergic agonist, clonazepam or GABAergic drugs.

The present study suggests a weak association between *HMOX1* rs2071746 polymorphism and the risk to develop RLS in the Spanish population.

## INTRODUCTION

Despite there are many data supporting the role of genetic factors in the aetiology and the pathogenesis of restless legs syndrome (RLS), also called Willis–Ekbom disease (WED), such as the high frequency of family history of RLS reported by patients with this syndrome, data found in family studies, and the higher concordance rates in monozygotic twins when compared with dizygotic ones, the identification of the responsible gene(s) remains to be clarified (revised in reference).^1^

The pathophysiology of idiopathic RLS is not well established. Although recent reports suggest the possible role of several neurotransmitters and/or neuromodulators such as aspartate, gamma-hydroxybutyric acid (GABA), glutamate and opiates, and a possible relation with vitamin D deficiency, the main hypotheses (likely interconnected) are dopaminergic dysfunction and iron deficiency (revised in reference).^2^

Heme oxygenase is an essential enzyme in heme catabolism, and it occurs as 2 main isozymes, an inducible heme oxygenase-1 (HMOX1) and a constitutive heme oxygenase-2 (HMOX2), which are encoded by the genes designated, respectively, as *HMOX1, HO-1* or *HSP32* (gene identity 3162, chromosome 22q13.1) and *HMOX2* or *HO-2* (gene identity 3163, chromosome 16p13.3). Several recent studies have shown association between certain single nucleotide polymorphisms (SNPs) in the *HMOX1*^3–6^ and *HMOX2*^5,7^ genes and the risk for Parkinson's disease (PD)^3–5,7^ and for essential tremor (ET).^6^

Because of the important role of brain iron deficiency in RLS, suggested by neuropathological, transcranial sonography, neuroimaging, and experimental data, it seems reasonable to study the possible association between SNPs related with iron metabolism and the risk for RLS. With the aim to investigate a possible association between *HMOX1* and *HMOX2* polymorphisms and the risk of developing RLS, we genotyped *HMOX1* and *HMOX2* SNPs in a large group of Caucasian Spanish RLS patients and controls.

## PATIENTS AND METHODS

### Patients and Controls

We studied 205 unrelated patients diagnosed with idiopathic RLS according to established RLS diagnostic criteria,^8,9^ and 445 gender-matched controls. Demographic data of both series are summarized in Table 1. Recruitment and diagnosis of RLS patients was carried out by consultant neurologists with expertise in Movement Disorders belonging to the Movement Disorders Units of 5 Hospitals. Inclusion criteria, besides the diagnosis of idiopathic RLS, were the absence of other previous neurological diseases, and the exclusion of possible causes of secondary RLS such as anaemia, renal failure, rheumatoid arthritis, peripheral neuropathy, and exposure to neuroleptics, antidepressants, or other drugs able to induce or to aggravate RLS. For this purpose, all patients underwent laboratory studies (blood count, routine biochemistry, iron metabolism studies, serum levels of vitamin B_12_, folic acid, and thyroid hormones, proteinogram, antinuclear antibodies, rheumatoid factor, and nerve conduction studies). The International RLS Study Group Rating Scale (IRLSSGRS)^10^ was used to assess RLS severity.

**TABLE 1:**
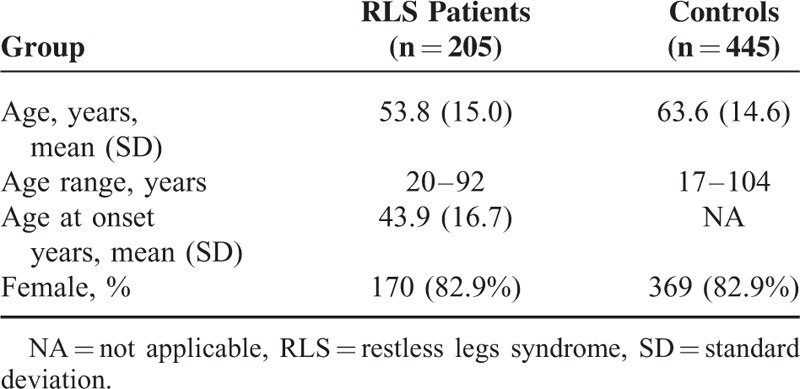
Demographic Data of the Series Studied

Positive family history of RLS was reported by 115 patients, 46 had ferritin levels between 30 and 50 ng/ml and 157 >50 ng/ml. Regarding previous treatments 159 have received dopamine agonists, 59 clonazepam, and 34 gabapentin or pregabaline, either alone or in combination.

The 445 controls were healthy Caucasian Spanish individuals matched by gender (275 of then were recruited from the Clínica Universitaria de Navarra, Pamplona, Spain; and the remaining 270 were recruited at the Infanta Cristina University Hospital, Badajoz, Spain). None of the controls had RLS, tremor, or other movement disorders.

### Ethical Aspects

All the participants included in the study gave their written informed consent after full explanation of the procedure. The Ethics Committees of Clinical Investigation of the Province of Cáceres (Cáceres, Spain), University Hospital “Príncipe de Asturias” (University of Alcalá, Alcalá de Henares, Madrid, Spain), the Infanta Cristina University Hospital (Badajoz, Spain), and Clínica Universitaria de Navarra (Pamplona, Spain) approved the study that was conducted according to the principles enumerated in the Helsinki Declaration of 1975. Most of the patients recruited had participated in other previous studies of genetic association with RLS risk.^11–14^

### Genotyping

Two single nucleotide polymorphisms in the *HMOX1* gene and two polymorphisms in the *HMOX2* gene were studied by means of TaqMan probes. Analyses included the *HMOX1* SNP rs2071746 (an upstream variant), *HMOX1* rs2071747 (a missense mutation within the exon 1 of the *HMOX1* gene), the SNP rs2270363 (a polymorphism in the regulatory region of the human *HMOX2* gene), and rs1051308 (a polymorphism in the 3′untranslated region). The selection of these SNPs was done because of their putative functional effects and their expected allele frequency in Caucasian individuals.^5,7^

Genotyping performed in genomic DNA from venous blood samples of participants and was carried out using TaqMan assays (Applied Biosciences Hispania, Alcobendas, Madrid, Spain) designed to detect the four previously mentioned SNPs designated respectively by the supplier with the following part numbers: C__15869717_10, C__22272778_10, C__15957370_10 and C___9695078_1_. An Eppendorf realplex thermocycler, using fluorescent probes, was used for the detection by qPCR. The amplification conditions were the following: after a denaturation time of 10 min at 96 °C, 45 cycles of 92 °C 15 s and 60 °C 90 s were carried out and fluorescence was measured at the end of each cycle and at endpoint. Determinations were done by triplicate in all samples, and then genotypes were assigned both using a gene identification software (RealPlex 2.0, Eppendorf) and analysing the reference cycle number for each fluorescence curve, calculated by means of the CalQPlex algorithm (Eppendorf).

The TaqMan copy number assays Hs00774483_cn and Hs01223070_cn, respectively, were used for the analysis of copy number variations (CNVs) of the *HMOX1* and *HMOX2* genes. Both assays were designed to hybridize within the open reading frame in the target genes (Applied Biosciences Hispania, Alcobendas, Madrid, Spain). An Applied Biosystems 7500 real-time thermocycler using as a copy number reference assay RNAse P was used to carry out the amplification, as described by the manufacturer. All reactions were carried out in quadruplicate. The analysis of the results were performed using the CopyCaller Software (Applied Biosciences Hispania). According to standard procedures in CNV analyses, we designed as heterozygous (null/present) those samples with a single copy of the corresponding gene. As the probes were designed to detect exonic sequences, even if the rest of the gene would remain in these so-called null alleles, the translated protein would not be functional.

### Statistical Analysis

The DeFinetti program (http://ihg.gsf.de/cgi-bin/hw/hwa1.pl) was used to analyze the Hardy–Weinberg equilibrium, the PLINK software^15^ to perform the allelic and genotype analyses, and the program PHASE v2.1.1^16^ to perform haplotype reconstruction using the default model for recombination rate variation with 1000 iterations, 500 burn-in iterations, and a thinning interval of 1 was used. Diplotypes were obtained from the combination of haplotypes in the best run (the one that showed the maximum consistency of results across all runs.^17^ Statistical analyses were performed using the SPSS 19.0 for Windows (SPSS Inc, Chicago, IL). We calculated intergroup comparison values by means of the χ2 or Fisher tests when appropriate, and calculated the 95% confidence intervals as well. The False discovery rate procedure^18^ was used to calculate correction for multiple testing (*Pc* values).

The determination of the sample size was done from variant allele frequencies observed in control individuals with a genetic model analyzing the frequency for carriers of the disease gene with a RR value = 1.5 (*P* = 0.05). The statistical power for 2-tailed associations for the presence of the SNPs identified in this study (rs2071746, rs2071747, rs2270363 and rs1051308) was 95.06%, 38.51%, 92.72% and 94.23%, respectively. The Breslow–Day test was used to analyze testing for heterogeneous association (homogeneity test).

The negative predictive value (NPV) was calculated as *d*/*r*2 (*d* = number of control individuals with the risk factor absent; *r*2 = sum of patients and controls with the risk factor absent).^19^

## RESULTS

The frequencies of the *rs2071746, rs2071747, rs2270363,* and *rs1051308* genotypes and allelic variants were in Hardy–Weinberg's equilibrium, both in RLS patient and control groups (Table 2). None of the patients and only 2 control individuals carried a single copy of the *HMOX1* and *HMOX2* genes, and hence CNVs were not further considered as major putative risk factors. The frequencies of *rs2071746TT* genotype and *rs2021746T* allele were significantly lower in RLS patients than in controls, both in the whole series (Table 2) and in female gender (Table 3). These differences remained significant for the rs2071746 allele frequency after multiple comparison analysis according to the false discovery rate correction. The frequencies of *rs2071747*, *rs2270363*, and *rs1051308* did not differ significantly between RLS patient and control groups.

**TABLE 2:**
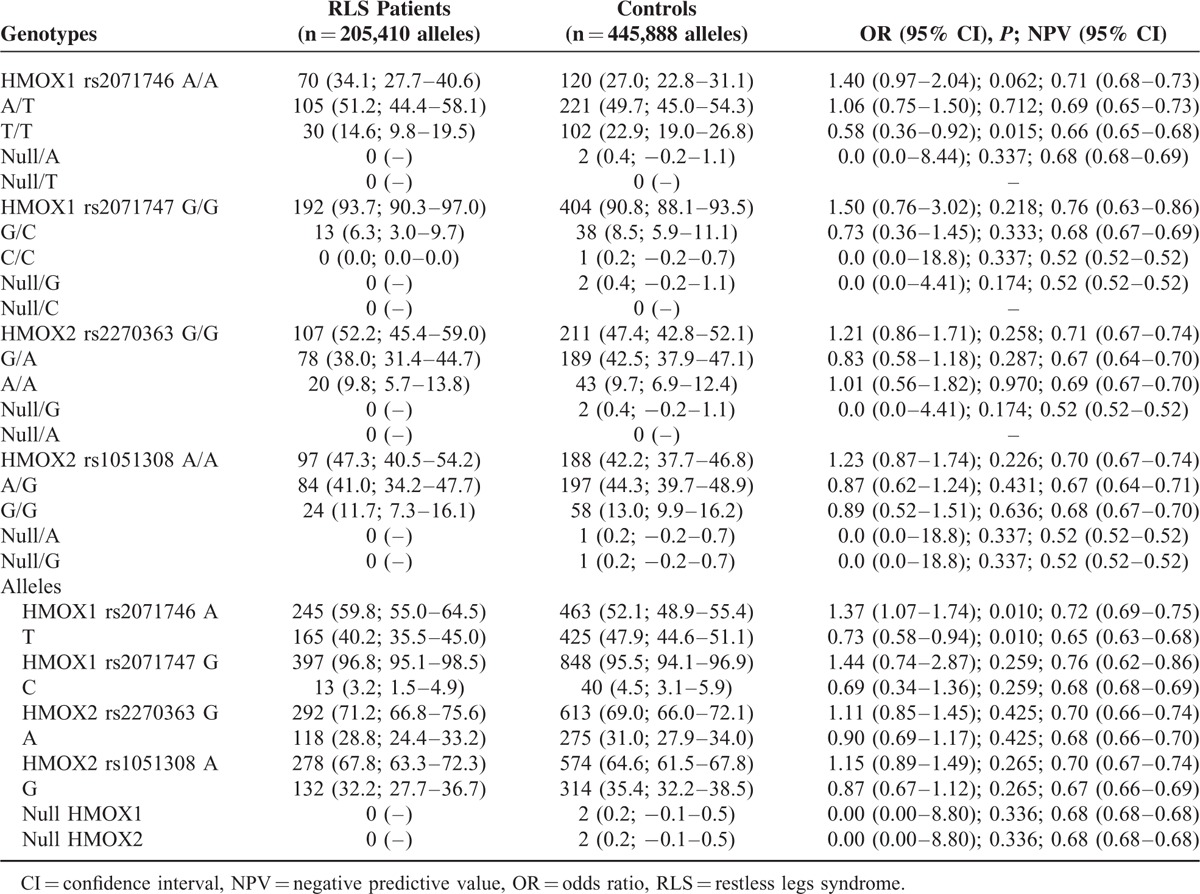
*HMOX* Genotypes and Allelic Variants of Patients with RLS and Healthy Volunteers. The Values in Each Cell Represent Number (Percentage; 95% Confidence Intervals)

**TABLE 3:**
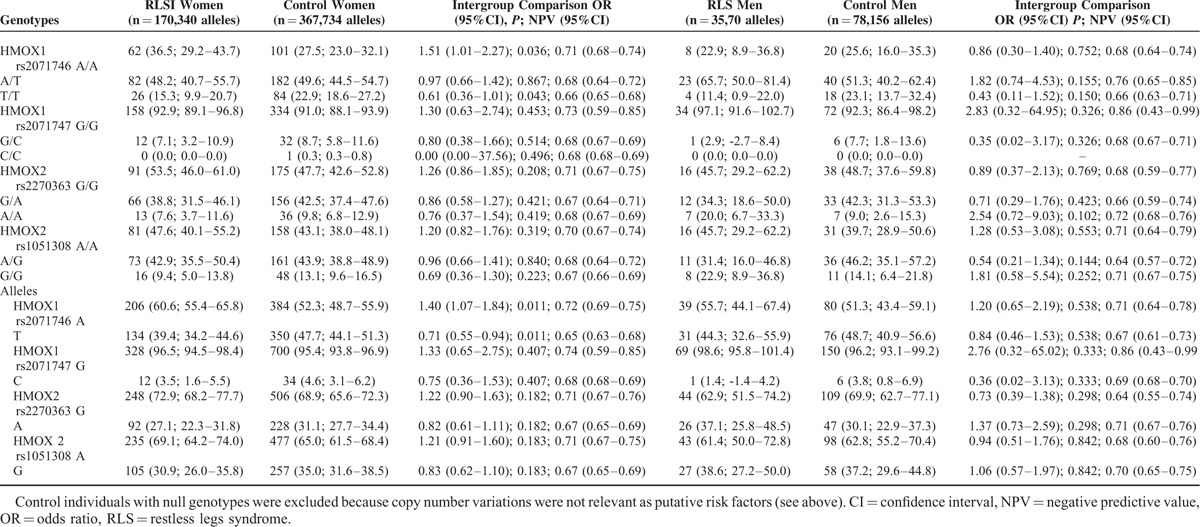
*HMOX* Genotypes and Allelic Variants of Patients with RLS and Healthy Volunteers Distributed by Gender. The Values in Each Cell Represent: Number (Percentage; 95% Confidence Intervals)

Mean + SD age at the onset of RLS did not differ between RLS patients carrying the genotypes *rs2071746AA, rs2071746AT,* and *rs2071746TT* (44.4 ± 16.6, 43.2 ± 17.9, and 44.4 ± 16.6 years, respectively); genotypes *rs2071747GG* and *rs2071747GC* (43.6 ± 17.0 and 43.8 ± 15.4 years, respectively); genotypes *rs2270363GG, rs2270363GA,* and *rs2270363AA* (43.4 ± 17.6, 44.2 ± 16.8, and 42.3 ± 13.1 years, respectively); and genotypes *rs1051308AA, rs1051308AG,* and *rs1051308AA* (42.9 ± 17.3, 45.0 ± 17.1, and 41.6 ± 14.5 years, respectively).

Mean + SD IRLSSGRS scores were similar for RLS patients carrying genotypes *rs2071746AA, rs2071746AT,* and *rs2071746TT* (24.6 ± 7.0, 25.02 ± 6.0, and 23.3 ± 8.0, respectively); genotypes *rs2071747GG* and *rs2071747GC* (24.8 ± 6.5 and 21.7 ± 6.4, respectively); genotypes *rs2270363GG, rs2270363GA,* and *rs2270363AA* (24.6 ± 6.8, 24.7 ± 6.6, and 22.7 ± 6.9, respectively); and genotypes *rs1051308AA, rs1051308AG,* and *rs1051308AA* (24.8 ± 6.7, 23.8 ± 6.6, and 23.8 ± 6.8, respectively).

The distribution of genotypes and allelic frequencies was similar in RLS patients with positive family history of RLS than in those with negative family history of RLS (Table 4), RLS patients with relatively low serum levels of ferritin (30–50 ng/ml) versus those with serum ferritin levels > 50 ng/ml (Table 4), and those RLS patients in which RLS symptoms improved with dopamine agonists, clonazepam, or gabapentin/pregabaline therapy compared with those who did not improve with these drugs (Table 5).

**TABLE 4:**
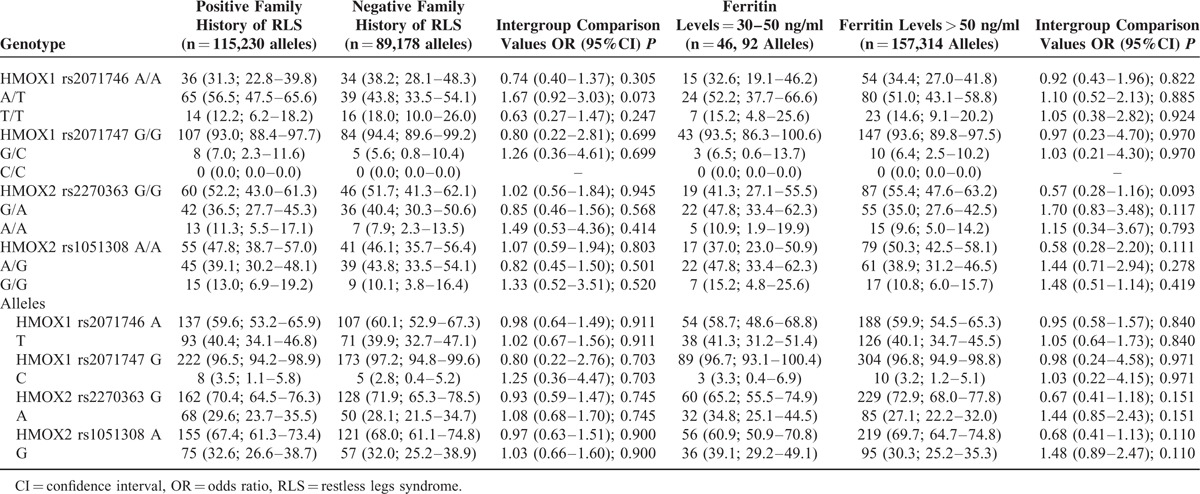
*HMOX* Genotypes and Allelic Variants of Patients with RLS Distributed by Family History and Serum Ferritin Levels. The Values in Each Cell Represent: Number (Percentage; 95% Confidence Intervals). Crude *P* Values are Shown

**TABLE 5:**
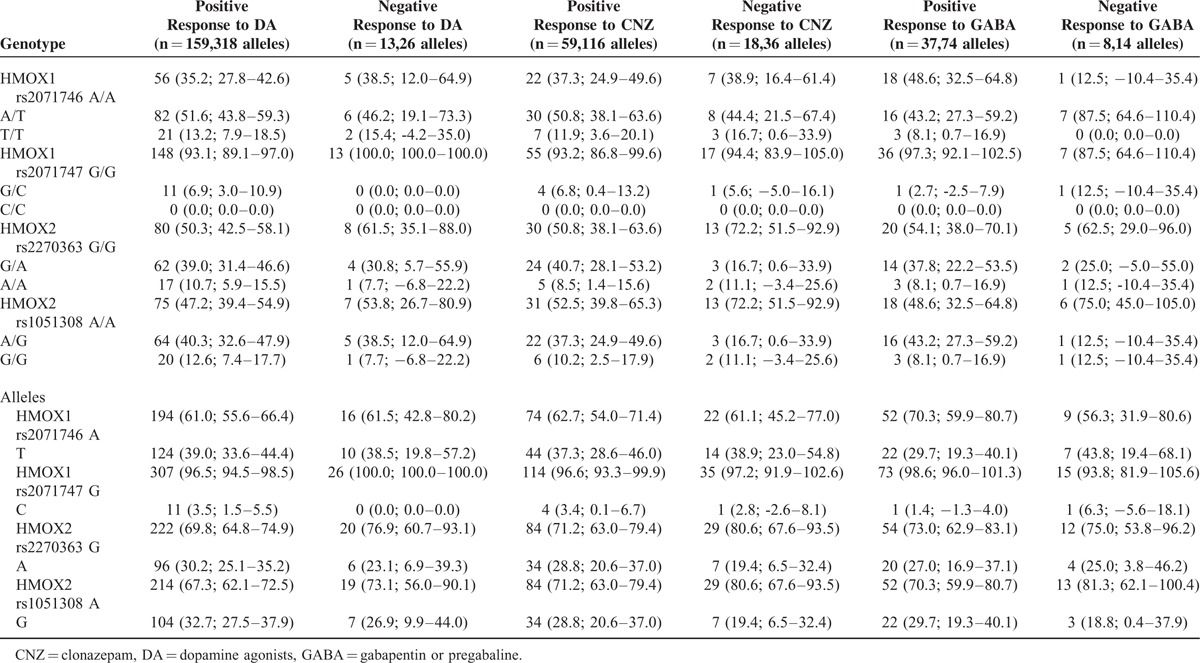
*HMOX* Genotypes and Allelic Variants of Patients with RLS Distributed by Drug Response. The Values in Each Cell Represent: Number (Percentage; 95% Confidence Intervals)

## DISCUSSION

Family reports of RLS are usually consistent with an autosomal dominant pattern of inheritance, although families with recessive or non-mendelian patterns have been described as well. To date, linkage studies have identified at least 8 genes/loci (most of them apparently autosomal dominant) in families with RLS. Association between several variants of *MEIS1* (closely related with iron metabolism),^20^*PTPRD,* and *BTBD9* genes and with the risk of developing RLS has been found in genome wide association studies (GWAS),^1^ whereas *PCDHA3* gene has been found to be related with the development of RLS in a large German family by using exome sequencing studies.^1^ Case-control association studies in RLS are scarce and inconclusive (revised in).^1^

Data from the present case-control association study suggest a weak association between the allelic variant *HMOX1 rs2071746T*, but not of the other 3 studied SNPs in the *HMOX*, and the risk for RLS. However, none of the studied SNPs were related with the age at onset or severity of RLS, positivity of family history or RLS, serum ferritin levels, and response to several treatments. The lack of relation between SNPs in the *HMOX* gene and serum ferritin levels is on the line of a previous study by Oexle et al,^21^ that described that SNPs at the known RLS loci and iron-related genes did not significantly affect serum iron parameters in several cohorts.

We have previously reported association between *HMOX1 rs2071746T* variant and the risk for PD and ET,^5,6^ suggesting a possible link between these diseases. However, how this SNP might change *HMOX* transcription both in PD (associated with elevated iron content) and RLS (associated with iron deficiency), and the possible putative mechanisms suggesting an association between HMOX and RLS could be speculative. So far there are no clues on putative biological mechanisms underlying the association found. The rs2071746 SNP is located in the 5′ area, ∼500 bp before the coding area, and therefore the most likely mechanism would be related to gene expression. The area where the SNP is located has several transcription factor binding sites. One of these, designated as CUTL1 [T00100] is present in the wild-type sequence, but disappears in the mutated sequence. The disruption of this transcription factor-binding site may underlie differences in gene expression.

In the brain, the HMOX pathway is a very important defensive mechanism against oxidative stress, mainly through the degradation of heme to biliverdin, free iron, and carbon monoxide. Moreover, an up-regulation of HMOX1 expression (resulting in increase of oxidative stress and sequestration of iron non-linked to transferrin in the mitochondrial department) has been found in the brains of patients with PD, Alzheimer's disease, and multiple sclerosis.^22,23^

Despite the pathophysiology of RLS is not well-established, it has been suggested an important role of iron deficiency in the pathogenesis of idiopathic RLS.^1^ Most of the magnetic resonance imaging studies found decreased iron content,^24–28^ and transcranial ultrasonography studies hypoechogenicity (reflecting decreased iron as well),^29–31^ in the substantia nigra of RLS patients compared with controls. Moreover, neuropathological studies have found decreased concentrations of iron, ferritin, and other proteins related with iron homeostasis in the substantia nigra of RLS patients.^32–36^ The results of studies assessing cerebrospinal fluid and serum/plasma levels of iron, ferritin, and transferrin are controversial (revised in).^2^ Several data in experimental models resembling RLS suggest an important interaction between iron deficiency and the dopaminergic system in the pathogenesis of RLS (revised in).^2^

To our knowledge, HMOX1 and HMOX2 expression has not been measured in neuropathological studies of RLS patients yet. It could be proposed that if the iron content is decreased in the substantia nigra of RLS patients, HMOX should act as protective against iron-related oxidative stress, and alterations in *HMOX1* and *HMOX2* genes could be related with the iron deficiency model of the pathogenesis of RLS.

A limitation of the present study is that, whereas 3 SNPs had a high statistical power, the other one, designated as rs2071747, had not. However, rs2071747 is an allele with a very low minor frequency (MAF) in healthy Europeans (0.045 in this study and 0.060 in the 1000 genomes database). Such SNP is also very rare in other human populations, MAF ranging from 0.020 to 0.050 (http://browser.1000genomes.org/Homo_sapiens/Variation/Population?db = core;r = 22:35776685–35777685;v = rs2071747;vdb = variation;vf = 1641286). To obtain a reliable statistical power for such SNP with the RR value = 1.5 (*P* = 0.05), the minimum sample size is estimated to be 1900 case-control pairs. However, it should be stressed that the significant findings in this study are related to another SNP that has a high statistical power: rs2071746 has a power equal to 95.6%.

Another potential limitation should be a selection bias. Although it is well known that in epidemiological studies the male-to-female ratios of incidence rate of RLS are in the range of 1:1.5 to 1:2.0 in adult populations, the male-to-female ratio of RLS patients is ∼1:5. This proportion is likely to be related with the fact that the recruitment of patients was done in a clinical (hospital-based) setting.

Although the results of the present study should be taken with caution because of the previously discussed limitations, and deserve further replication studies in other populations, they suggest a slightly decreased risk for RLS in Spanish Caucasian individuals carrying the *HMOX1 rs2021746T* allele variant.
